# Late Gastrointestinal Tolerance After Prostate Radiotherapy: Is the Anal Canal the Culprit? A Narrative Critical Review

**DOI:** 10.3389/fonc.2021.666962

**Published:** 2021-06-16

**Authors:** Paul Sargos, Mame Daro Faye, Manon Bacci, Stéphane Supiot, Igor Latorzeff, David Azria, Tamim M. Niazi, Te Vuong, Véronique Vendrely, Renaud de Crevoisier

**Affiliations:** ^1^ Department of Radiation Oncology, Institut Bergonié, Bordeaux Cedex, France; ^2^ Department of Radiation Oncology, Jewish General Hospital, Montreal, QC, Canada; ^3^ Department of Radiation Oncology, Institut de Cancérologie de l’Ouest, Saint-Herblain, France; ^4^ Department of Radiation Oncology, Clinique Pasteur, Toulouse, France; ^5^ Department of Radiation Oncology, Institut de Cancérologie de Montpellier, Montpellier, France; ^6^ Department of Radiation Oncology, Bordeaux University Hospital, Bordeaux, France; ^7^ Department of Radiation Oncology, Centre Eugene Marquis, Rennes, France

**Keywords:** prostate cancer, radiotherapy, gastrointestinal toxicities, anal canal, rectum

## Abstract

**Introduction:**

Late gastro-intestinal toxicities (LGIT) secondary to pelvic radiotherapy (RT) are well described in the literature. LGIT are mainly related to rectal or ano-rectal irradiation; however, involvement of the anal canal (AC) in the occurrence of LGIT remains poorly described and understood.

**Materials and Methods:**

The aim of this work was to explore the potential role of the AC in the development of LGIT after prostate irradiation and identify predictive factors that could be optimized in order to limit these toxicities. This narrative literature review was realized using the Pubmed database. We identified original articles published between June 1997 and July 2019, relating to LGIT after RT for localized prostate cancer and for which AC was identified independently. Articles defining the AC as part of an anorectal or rectal volume only were excluded.

**Results:**

A history of abdominal surgery or cardio-vascular risk, anticoagulant or tobacco use, and the occurrence of acute GIT during RT increases the risk of LGIT. A dose-effect relationship was identified between dose to the AC and development of LGIT. Identification and contouring of the AC and adjacent anatomical structures (muscles or nerves) are justified to apply specific dose constraints. As a limitation, our review mainly considered on 3DCRT which is no longer the standard of care nowadays; we did not identify any reports in the literature using moderately hypofractionated RT for the prostate and AC specific dosimetry.

**Conclusion:**

These results suggest that the AC may have an important role in the development of LGIT after pelvic RT for prostate cancer. The individualization of the AC during planning should be recommended in prospective studies.

## Introduction

Gastrointestinal toxicities (GIT), acute or late (LGIT), can occur after radiotherapy (RT) for localized prostate cancer, altering the quality of life of up to 50% of patients (1, 2). These LGIT include gastrointestinal bleeding, painful bowel movements, increased stool frequency, diarrhea or mucous in stool, fecal urgency, incontinence, as well as abdominal pain (3–5). Matta et al. (6) recently reported that grade >2 LGIT occur in 2% to 26% of patients treated with conventional fractionation prostate RT (dose level between 70 and 80 Gy), whereas for moderately hypofractionated RT, the rate of grade >2 LGIT is lower than 6% (6). In the case of ultra hypofractionated prostate RT (over 6 Gy per fraction), grade >2 LGIT rates are between 0% and 4% (6). Thus, it appears that evolving RT techniques allow for a better tolerance of dose escalation treatment regimens.

LGIT are often thought to be related to irradiation of organs at risk (OARs), such as the rectum, the ano-rectal volume, and/or the small bowel. However, there is no clear correlation between irradiation of the anal canal (AC) alone and the development of LGIT (7). Indeed, the long-term effects of pelvic RT on the function of the rectum or the ano-rectum have been largely studied, with a specific focus on GIT (8–13). However, these studies did not identify the AC independently despite it being often included in a non-specific OAR volume (8–13). Pelvic irradiation could lead to certain toxicities, some of which could be specific to the AC (14, 15).

The CTCAE classification is a descriptive scale for the severity of symptoms, graded from 1 to 5, which could arise from any treatment. In its fourth version (v4.0) (14), toxicities described for the AC include anal fistulas, anal hemorrhage, mucositis, ulceration, necrosis, stenosis, and anal pain. However, symptoms related to fecal incontinence, diarrhea, and hemorrhoids are not directly attributed to irradiation of the AC, but rather to the GI structures in general (14). The same observation can be made in other classification systems used to describe radiation-related gastrointestinal toxicities, such as the Radiation Therapy Oncology Group/European Organization for Research and Treatment of cancer (RTOG/EORTC) (16–18) or the subjective-objective-management-analytic–late effects of normal tissues (SOMA-LENT) (8, 19–24). This prompted us to do a literature review to assess the individual contribution of AC irradiation on the incidence of LGIT after prostate RT.

The objectives of this narrative critical literature review were to explore if LGIT could be potentially related to irradiation of the AC in the context of prostate cancer RT. We sought to analyze the extent to which the AC is involved in the development of radiation-related LGIT, to bring to light different predictive factors for the occurrence of LGIT and to show a potential dose-effect relationship between dose to the AC and the development of LGIT. The overall goal is to optimize the care and quality of life of patients undergoing prostate RT.

## Materials and Methods

### Literature Search

This narrative literature review was realized using the Pubmed database. Articles published in any language between June 1997 and July 2019 were included. The PICO research question was “Is irradiation of the anal canal as an individual structure related to the development of LGIT in prostate cancer patients treated with radiotherapy” (P: prostate cancer patients treated with radiotherapy; I: irradiation of the anal canal as an individual volume; C: none; O: LGIT). Keywords for the search included « anal canal », « anorectal » « prostate », « Prostatic Neoplasms/radiotherapy », « gastrointestinal », « toxicity », « NTCP » and « probability », used separately or in combination. Bibliographic references cited within eligible articles could be used as well. Eligible articles were ranked and prioritized based on quality of the publication starting with randomized control trials (RCTs), then prospective studies, and finally retrospective studies, but given the limited number of articles found, they were all considered in this review.

### Inclusion and Exclusion Criteria

All selected articles discussed on the RT of localized prostate cancer with or without pelvic lymph nodes irradiation. All cases were biopsy-proven adenocarcinoma of the prostate, localized (T1-T4) and non-metastatic. We selected the articles where LGIT were clearly cited as a primary or secondary objective of the study. GIT were defined as late when they occurred more than 6 months after the end of RT. Selected articles also had to contain an analysis of the toxicities related to the AC, defined as a specific OAR, meaning that articles evaluating LGIT on a rectal or ano-rectal volume only were out of the scope of our review. Exclusion criteria were: articles on acute toxicities only, late GIT not specifically related to the AC, i.e., not specifically describing AC-related symptoms and/or grade, articles that did not specify the AC as a separate OAR, articles on late GIT related to pelvic irradiation but for other cancer histologies, letters to the editors, commentaries, and abstracts.

### Articles Selection

Sixty-three articles were identified using the above search method. One abstract, one editorial, and two letters were excluded. Thirty additional articles were excluded based on their titles. The abstracts of the 30 remaining articles were read independently by two authors and those not specifically related to the AC-related late GIT or the AC as an OAR were excluded, leaving a total of nine articles for further analysis. Bibliographic references cited in these nine papers are also cited when contributory to the discussion or for background information.

## Results of the Literature Search

The majority of the articles from our literature search were on LGIT after prostate RT, however only nine articles were specifically on the AC and met all our inclusion and exclusion criteria (16, 17, 23, 25–30).

### Impact of Anal Canal Irradiation on Rectorrhagia in the Context of Pelvic Radiotherapy

After treatment of prostate cancer with RT, the risk of grade ≥ 2 rectorrhagia specifically related to irradiation of the AC is between 5% and 10% (31, 32). Identification of clinical and dosimetric predictive factors related to GI bleeding after prostate RT has been the subject of many publications (16, 17, 19–24, 27, 28, 33). These studies looked at patients that were specifically treated to the prostate with three-dimensional conformal radiation therapy (3D-CRT), at doses between 64 and 78 Gy. The studies that specifically defined the AC as an OAR are presented in **Table 1**.

**Table 1:** Identification of predictive factors for rectorrhagia in patients with localized prostate cancer treated with pelvic radiotherapy.

Study	Design	No. of patients	Received treatment (technique, dose, volumes and associated treatments)	Follow-up (months)	Outcomes/Endpoint definition	Evaluation method	Predictive factors identified in multivariate analysis
Peeters et al. (16)	Multicenter phase 3 RCT	641	3D Dose: 68 vs 78 Gy CTV1= prostate only; CTV2= prostate + SV up to 50 Gy then prostate only; CTV3= same as CTV3, treated to 68 Gy; CTV4= prostate + SV treated to total dose PTV= 10 mm margin coned down to 5 mm for delivery of the last 10 Gy (in 78 Gy arm) Anal canal = most distal 3 cm of the anorectal volume HT for grade group 3 and 4	44	Grade ≥ 2 rectal bleeding, requiring treatment by laser coagulation and/or blood transfusion	Modified RTOG/EORTC score	1. History of abdominal surgery (HR =2.7; p ≤ 0.01)
Ebert et al. (27)	Multicenter phase 3 RCT	754	3D Dose: 66, 70, 74 or 78 Gy CTV= prostate only +/− SV; PTV= 10 mm margin reduced to 5 mm posteriorly Anal canal = most distal 3 cm of the anorectal volume HT for 6 vs 18 months	72	Prevalence of peak toxicity grade at 36 months, sometimes requiring iron supplements	SOMAT LENT and CTCAE V2.0	1. V40 Gy to V65 Gy was predictive of anorectal bleeding ay 36 months
Schaake et al. (30)	Prospective cohort	262	IMRT Dose: 78 Gy CTV= prostate only +/− SV; PTV= 10 mm isotropic margin Pelvic floor muscles were contoured retrospectively Anal canal = most distal 3 cm of the anorectal volume HT was allowed	> 36 months	Prevalence of grade ≥2 toxicities after 36 months	CTCAE V3.0 and patient questionnaire	1. The use of anticoagulants increases the risk of rectorrhagia (OR=3 ; p=0.06)
Defraene et al. (28)	Phase 3 RCT	512	3D Dose: 68 vs 78 Gy CTV1= prostate only; CTV2= prostate + SV up to 50 Gy then prostate only; CTV3= same up to 68Gy; CTV4= prostate + SV for total dose PTV= 10 mm margin coned down to 5 mm for delivery of the last 10 Gy (in 78 Gy arm) Anal canal = most distal 3 cm of the anorectal volume HT for grade groups 3 and 4	> 36 months	Prevalence of the critical event	Subjective	History of abdominal surgery or cardiovascular risk/disease V65Gy to the anal canal (p=0.002)

RCT, randomized controlled trial; 3D, tridimensional radiotherapy; RT, radiotherapy; CTV, clinical target volume; PTV, Planning Target Volume, SV, seminal vesicles; H, hormonotherapy; OR, odds ratio; HR, hazard ratio.

Only one study, the TROG 03.04 RADAR trial that analyzed LGIT after prostate RT using the SOMA-LENT scale with a median follow-up of six years, showed the dose-effect relationship of moderate to high doses of RT to the AC (> 40 Gy) and rectorrhagia incidence at 36 months (27). In this study, V40 Gy to V65 Gy to the whole anorectum was predictive of anorectal bleeding whereas low-to-mid doses to the AC were predictive of rectorrhagia. Interestingly, the peak incidence of rectorrhagia attributed to the rectum peaked before 24 months whereas for the AC, it peaked at 36 months. Thus, the authors suggested an association of earlier bleeding with dose to the rectum and delayed bleeding with dose to the AC dose (27).

Peeters et al. (16) showed that rectorrhagia needing treatment correlated most strongly with anorectal V55–V65 Gy (p < 0.01), with the most significant parameter being V65 (p < 0.004). Interestingly, on multivariate analysis, rectal parameters were slightly less significant for rectorrhagia compared with the corresponding anorectal variables (16). Then, delineation of a separate AC subvolume in future prospective studies may help tease out the different contributions of the rectal and AC structures to late rectorrhagia (33, 34).

As for patient-related factors, it appears that a history of abdominal surgery (16, 19, 20, 28) increases the risks of rectorrhagia after pelvic irradiation. Peeters et al. (16) showed that RTOG/EORTC Grade ≥2 toxicities were significantly associated with low to intermediate dose anal parameters and anal Dmean. Importantly, adding the variables of abdominal surgery and pretreatment GI symptoms increased the level of significance of this association on multivariate analysis. The same authors showed that including a history of abdominal surgery to the Lyman Kutcher Burman (LKB) model, which also includes dose to the AC, improved its prediction capacity for late rectal bleeding (17). The TD50 (dose at which toxicity occurs in 50% of the population) was estimated at 81 Gy using the original LKB model. However, using their modified stratified model, TD50 was 85 Gy in patients without any past abdominal surgery history compared to 78 Gy for patients with a history of abdominal surgery (16, 17). These results were corroborated by Defraene et al. who also showed that cardiovascular history was predictive of LGIT (28).

### Impact of Anal Canal Irradiation on Stool Frequency and Diarrhea in the Context of Pelvic Radiotherapy

The risk of developing grade ≥ 2 diarrhea and increased stool frequency after prostate RT varies between 4% and 19% (24). **Table 2** summarizes the AC dosimetric data and patient-related factors that are predictive of increased stool frequency and diarrhea. The TROG 03.04 RADAR study, in which the AC was individually defined as an OAR, showed that low to moderate doses to the AC (4–38 Gy) were correlated with increased stool frequency (27). Interestingly, in another study, an increase in stool frequency was associated with dose to pelvic muscles such as the iliococcygeal muscle (V45) and the levator ani (V40) (30).

**Table 2:** Identification of predictive factors for the late occurrence of diarrheas and increased stool frequency in patients with localized prostate cancer treated with pelvic radiotherapy.

Study	Design	No. of patients	Received treatment (technique, dose, volumes and associated treatments)	Follow-up(months)	Outcomes/Endpoint definition	Evaluation method	Predictive factors identified in multivariate analysis
Peeters et al. (16)	Multicenter phase 3 RCT	641	3D Dose: 68 vs 78 Gy CTV1= prostate only; CTV2= prostate + SV up to 50 Gy then prostate only; CTV3= same as CTV3, treated to 68 Gy; CTV4= prostate + SV treated to total dose PTV= 10 mm margin coned down to 5 mm for delivery of the last 10 Gy (in 78 Gy arm) Anal canal = most distal 3 cm of the anorectal volume HT for grade groups 3 and 4	44	≥ 6 bowel movements/day	Modified RTOG/EORTC score	1. History of acute GIT (p ≤ 0,01, HR 2,9)
Peeters et al. (17)	Multicenter phase 3 RCT	468	3D Dose: 68 vs 78 Gy CTV1= prostate only; CTV2= prostate + SV up to 50 Gy then prostate only; CTV3= same as CTV3, treated to 68 Gy; CTV4= prostate + SV treated to total dose PTV= 10 mm margin coned down to 5 mm for delivery of the last 10 Gy (in 78 Gy arm) Anal canal = most distal 3 cm of the anorectal volume HT for grade groups 3 and 4	36	≥ 6 bowel movements/day	Modified RTOG/EORTC score	1. Inclusion of clinical factors, such as a history of abdominal surgery and acute GIT, into a modified LKB (Lyman-KutcherBurman) model significantly improves the prediction of complications
Ebert et al. (27)	Multicenter phase 3 RCT	754	3D Dose: 66, 70, 74 or 78 Gy CTV= prostate only +/− SV; PTV= 10 mm margin reduced to 5 mm posteriorly Anal canal = most distal 3 cm of the anorectal volume HT for 6 vs 18 months	72	Prevalence of peak toxicity grade at 36 months	SOMAT LENT and CTCAE V2.0	1. Low to moderate radiotherapy doses (4 to 8 Gy)
Schaake et al. (30)	Prospective cohort	262	IMRT Dose: 78 Gy CTV= prostate only +/− SV; PTV= 10 mm isotropic margin Pelvic floor muscles were defined retrospectively Anal canal = most distal 3 cm of the anorectal volume HT was allowed	> 36 months	Prevalence grade ≥2 toxicities after 36 months	CTCAE V3.0 and patient questionnaire	Dmean ICM, Dmean PRM and D mean LAM ICM: V45 Gy LAM: V40 Gy
Defraene et al. (28)	Phase 3 RCT	512	3D Dose: 68 vs 78 Gy CTV1= prostate only; CTV2= prostate + SV up to 50 Gy then prostate only; CTV3= same up to 68Gy; CTV4= prostate + SV for total dose PTV= 10 mm margin coned down to 5 mm for delivery of the last 10 Gy (in 78 Gy arm) Anal canal = most distal 3 cm of the anorectal volume HT for grade groups 3 and 4	> 36 months	Prevalence of the critical event	Subjective	1. History of increased stool frequency (>3 per day) before radiotherapy

RCT, randomized controlled trial; 3D, tridimensional radiotherapy; RT, radiotherapy; CTV, clinical target volume; PTV, planning target volume; SV, seminal vesicles; HT, hormonotherapy; GIT, gastrointestinal toxicities; OR, odds ratio; HR, hazard ratio; IAS, internal anal sphincter; EAS, external anal sphincter; PRM, puborectal muscle; ICM, iliococcygeal muscle; PRM+ICM=LAM, levator ani.

As for patient-related predictive factors, a history of previous abdominal surgery is related to an increased risk in stool frequency and in diarrhea after prostate RT (17). Other studies (16, 17, 28) have also shown that the presence of acute GI side effects during RT or a history of GI symptoms prior to irradiation are both risk factors for increased stool frequency and diarrhea later on.

### Impact of Anal Canal Irradiation on Renesmus, Stool Urgency, and Incontinence in the Context of Pelvic Radiotherapy

The risk of chronic stool urgency and tenesmus (grade ≥2) after prostate RT varies between 3% and 12%. Stool incontinence is reported to be around 5% in most series; however, it greatly affects patients’ quality of life (19), hence, the need to find dosimetric and patient-related factors than can predict which patients are more at risk of developing stool incontinence. **Table 3** summarizes some of these factors related to the AC.

**Table 3:** Identification of predictive factors for tenesmus, stool urgency or incontinence in patients with localized prostate cancer treated with pelvic radiotherapy.

Study	Design	No. of patients	Received treatment (technique, dose, volumes and associated treatments)	Follow-up(months)	Outcomes/Endpoint definition	Evaluation method	Predictive factors identified in multivariate analysis
Peeters et al. (16)	Multicenter phase 3 RCT	641	3D Dose: 68 vs 78 Gy CTV1= prostate only; CTV2= prostate + SV up to 50 Gy then prostate only; CTV3= same as CTV3, treated to 68 Gy; CTV4= prostate + SV treated to total dose PTV= 10 mm margin coned down to 5 mm for delivery of the last 10 Gy (in 78 Gy arm) Anal canal = most distal 3 cm of the anorectal volume HT for grade groups 3 and 4	44	- Grade ≥ 2 rectal bleeding requiring treatment by laser coagulation and/or blood transfusion	Modified RTOG/EORTC score	V5-70 Gy, Dmean (p=0.002) and V65 (p=0.0004) are predictive of incontinence. The incidence of stool incontinence is < 10% if Dmean is < 46 Gy A history of acute GIT is predictive of stool incontinence (HR =1.9; p ≤ 0.01) A history of abdominal surgery is predictive of stool incontinence (HR= 2.2; p ≤ 0.01)
Peeters et al. (17)	Multicenter phase 3 RCT	468	3D Dose: 68 vs 78 Gy CTV1= prostate only; CTV2= prostate + SV up to 50 Gy then prostate only; CTV3= same as CTV3, treated to 68 Gy; CTV4= prostate + SV treated to total dose PTV= 10 mm margin coned down to 5 mm for delivery of the last 10 Gy (in 78 Gy arm) Anal canal = most distal 3 cm of the anorectal volume HT for grade groups 3 and 4	36	≥ 6 bowel movements /day	Modified RTOG/EORTC score	1. A history of abdominal surgery specifically if low) increases the risk of all late anal canal RT- related toxicities
Ebert et al. (27)	Multicenter phase 3 RCT	754	3D Dose: 66, 70, 74 or 78 Gy CTV= prostate only +/− SV; PTV= 10 mm margin reduced to 5 mm posteriorly Anal canal = most distal 3 cm of the anorectal volume HT for 6 vs 18 months	72	Prevalence of peak toxicity grade at 36 months	SOMAT LENT and CTCAE V2.0	1. Low to moderate RT doses (5 to 38 Gy) increase the risk of tenesmus and urgency
Schaake et al. (30)	Cohort prospective	262	IMRT Dose: 78 Gy CTV= prostate only +/− SV; PTV= 10 mm isotropic margin Pelvic floor muscles were defined retrospectively Anal canal = most distal 3 cm of the anorectal volume HT was allowed	> 36 months	Prevalence of grade ≥2 toxicities after 36 months and diaper/pads use	CTCAE V3.0 and patient questionnaire	Dmean to all pelvic muscles is predictive of stool incontinence EAS: V15 Gy is predictive of incontinence ICM: V55 Gy is predictive of incontinence
Thor et al. (29)	Prospective cohorts	212 in the Danish cohort 277 in the Swedish cohort	3DCRT Dose: 70 to 78 Gy CTV= prostate only +/− SV PTV= For the Danish cohort, 7 mm margin but 9 mm cranio-caudally. For the Swedish cohort, 20 mm margin but 15 mm posteriorly Definition of the anal sphincter (AS) and of external/internal sphincter muscles Definition of anal sphincter (AS)	42 months for the Danish cohort 76 months for the Swedish cohort	Prevalence of moderately severe symptoms (occurring at least once/week) and the use of diapers/pads	Questionnaires specific to the Danish and Swedish cohorts	Tobacco, Dmin and low RT doses are predictive of stool urgency Age, tobacco, follow-up length and low RT doses (D100, D95, and V30 Gy) are predictive of stool incontinence V70 Gy is predictive of tenesmus
Buettner et al. (23)	Multicenter phase 3 RCT(MRC RT01)	388	3DCRT Dose: 64 Gy vs 74 Gy CTV= prostate +/− SV PTV= 10 mm margin. No additional margin for the 74 Gy group Anal canal defined as the last, most distal 3 cm of the rectum	120 months	Highest toxicity grade score and use of pads	Graded scale defining 7 clinically significant symptoms	Lateral extension of the dose at 53 Gy beyond 56% is predictive of sphincteric control Dmean >45.1 Gy is predictive of sphincteric control Dmean >47 Gy is predictive of sphincteric control No predictive factors for the other GI symptoms
Smeenk et al. (25)	Prospective controlled trial	90	3D or IMRT Dose: 67.5 to 70Gy in 2.25 to 2.50 Gy fractions CTV= prostate +/− SV No details provided on the PTV Retrospective delineation of the rectum and anal canal Some patients were treated with an ERB	≥ 24 months	Presence or absence of symptoms.	RILIT	Urgency: Significant decrease in symptoms if reduction of the Dmin (10.1 vs 4.9 Gy, p = 0.04), Dmean (42.1 vs 31.6 Gy, p=0.02), and of V30, V40, V50, V60 Gy anal Incontinence: Significant decrease in symptoms if reduction of the Dmin (10 vs 5 Gy; p=0.04) and of V50 (33 vs 20Gy; p=0.04) anal
Smeenk et al. (26)	Observational study	48	3D of IMRT Dose: 67.5 to 70Gy in 2.25 to 2.50 Gy fraction CTV= prostate +/− SV No details provided on the PTV Retrospective delineation of the anal and rectal wall, puborectal muscle (PRM), levator ani muscles (LAM), internal (IAS) and external (EAS) sphincter muscles Some patients were treated with an ERB	24 to 30 months	Presence or absence of symptoms	RILIT	Urgency: Dmean<30 Gy IAS, Dmean<10 Gy EAS, Dmean <50 Gy PRM and Dmean<40 Gy LAM reduce the risk of stool urgency. Dmax EAS=50Gy (p=0.001), Dmin PRM=23.8 (p=0.001) and LAM=25.2 (p=0.02) are predictive of urgency. Dmean=30 (p=0.04) as well as V20, 30, 40, 50 and 60 Gy are also predictive. Incontinence: Increasing the Dmax=51.5 Gy (p=0.009), Dmean EAS=16.5 Gy (p=0.005) and DminPRM=24.8 Gy (p=0.03) are predictive of stool incontinence
Defraene et al. (28)	Phase 3 RCT	512	3D Dose: 68 vs 78 Gy CTV1= prostate only; CTV2= prostate + SV up to 50 Gy then prostate only; CTV3= same up to 68Gy; CTV4= prostate + SV for total dose PTV= 10 mm margin coned down to 5 mm for delivery of the last 10 Gy (in 78 Gy arm) Anal canal = most distal 3 cm of the anorectal volume HT for grade groups 3 and 4	> 36 months	Prevalence of the critical event	subjective	1. V30 of the anal canal (p=0.004) History of abdominal surgery (p<0.001) and diabetes (p=0.05)

RCT, randomized controlled trial; 3D, tridimensional radiotherapy; RT, radiotherapy; CTV, clinical target volume; PTV, planning target volume; SV, seminal vesicles; HT, hormonotherapy; GIT, gastrointestinal toxicities; OR, odds ratio; HR, hazard ratio; IAS, internal anal sphincter; EAS, external anal sphincter; PRM, puborectal muscle; ICM, iliococcygeal muscle; PRM+ICM=LAM, levator ani; ERB, endorectal balloon.

Low to moderate doses of RT to the AC were associated with increased risk of tenesmus, stool urgency, and incontinence to the same extent as higher doses. Indeed, Peeters et al. (16) showed that all dosimetric parameters to the anal wall were significantly predictive of stool incontinence. Smeenk et al. (25) found a significant decrease in AC rest pressure in patients presenting with stool urgency and incontinence. Moreover, AC Dmin, Dmean, V30, V40, V50, and V60 of the AC were all correlated with the incidence of stool urgency and incontinence in this study (25). The V30 Gy and a dose of 5 to 38 Gy to the AC were also identified as predictive factors in an NTCP model by Defraene et al. (28). In a multicenter RCT of 388 patients, with a median follow-up of 24 months, Buettner et al. (23) established a significant positive correlation between sphincter-related symptoms and the dose received specifically by the AC wall. The authors recommended mean doses of 30 Gy or less to the anal sphincter and of 27 Gy at most to the AC surface to limit the risks of tenesmus, stool urgency and incontinence.

The frequency, intensity, and chronicity of LGIT have been significantly correlated with manometric studies of anal pressures (8–12). Some of these studies showed a change in the morphology of the internal and external sphincters, whereas others did not, underlying a possible, but not proven, contribution of the AC in the occurrence of lower LGIT related to prostate RT. This relation between the tissue response of anal structures to RT and anorectal dysfunction is further supported by a cohort study of 309 patients with prostate cancer treated by RT (10). With a median follow-up of 3.8 years, the patients with high RT-induced anorectal dysfunction had changes to the anorectal mucosa, increased rectal sensory response to distension, and reduced maximum anal resting pressure as assessed by anal manometri (10). Altogether, these findings suggest a correlation between RT-induced LGIT and RT-induced morphologic changes to the AC. They also suggest a specific role for the AC in the development of these LGIT.

Irradiation of the pudendal nerve, which is closely related to the AC, was also implicated in the development of stool incontinence. In a retrospective study, 17 patients with localized prostate cancer were treated by RT whereas a control group of 57 patients were not (35). The authors described a loss of response of the pudendal nerve to stimulation in 10 patients treated with RT (62.5%) vs 3 patients (6.5%) in the control group (p< 0,001). Moreover, there was altered pudendal nerve response in four patients treated with RT to the prostate (25.1%) vs seven (15.2%) in the control group (p< 0,001). It would be interesting to see if spatial extension of the dose to the AC played a role in the occurrence of these LGIT, as was shown for the rectum (34). Finally, radiation doses to the pelvic floor muscles and anal sphincters have been shown to have an impact on the occurrence of stool urgency and incontinence (26).

As for patient-related predictive factors, once again, a history of abdominal surgery was a risk factor for the development of tenesmus, stool urgency, and incontinence (16, 17, 21, 22, 24, 28) whereas the use of anti-hypertensive medications seemed protective (21, 22). Interestingly, the “lower” the level of the abdominal surgery was, the more the risk of such LGIT. One hypothesis to explain this is the fact that abdominal surgery causes a state of inflammation prior to RT, with an increased production of cytokines (20, 36, 37). Furthermore, surgery alters the neurovascular system in the area of concern, leading to higher sensitivity to radiation (17). Finally, it appears that diabetic patients are more at risk as well (28), probably through the same inflammatory processes.

### Impact of AC Irradiation on Gastrointestinal and Abdominal Pain in the Context of Pelvic Irradiation

There is scarce data available on this type of symptom and their frequency after prostate RT. It seems however that less than 5% of patients present with grade ≥ 2 late gastrointestinal or abdominal pain (16, 24, 38) (**Table 4**). Peeters et al. (16) showed that the presence of acute GIT during RT or a history of GI symptoms prior to irradiation significantly increases the incidence of gastrointestinal and abdominal pain later. According to a study by Thor et al., tobacco smoking and hormonotherapy are contributing factors, whereas the V15 of the AC could be predictive of late GI or abdominal pain (29). However, no anal or rectal dosimetric parameters were shown to be directly related to these symptoms.

**Table 4:** Identification of predictive factors for late abdominal or rectal pain in patients with localized prostate cancer treated with pelvic radiotherapy.

Study	Design	No. of# patients	Received treatment (technique, dose, volumes and associated treatments)	Follow up(months)	Outcomes/Endpointdefinition	Evaluation method	Predictive factors identified in multivariate analysis
Peeters et al. (16)	Multicenter phase 3 RCT	641	3D Dose: 68 vs 78 Gy CTV1= prostate only; CTV2= prostate + SV up to 50 Gy then prostate only; CTV3= same as CTV3, treated to 68 Gy; CTV4= prostate + SV treated to total dose PTV= 10 mm margin coned down to 5 mm for delivery of the last 10 Gy (in 78 Gy arm) Anal canal = most distal 3 cm of the anorectal volume HT for grade groups 3 and 4	44	Grade ≥ 2 rectal bleeding, requiring treatment by laser coagulation and/or blood transfusion	Modified RTOG/EORTC score	1. Acute GIT (HR=1.9; p ≤ 0.01) 2. GI symptoms prior to radiotherapy 2. No association found with dosimetric parameters (HR=6.4; p ≤ 0.01)
Ebert et al. (27)	Multicenter phase 3 RCT	754	3D Dose: 66, 70, 74 or 78 Gy CTV= prostate only +/− SV; PTV= 10 mm margin reduced to 5 mm posteriorly Anal canal = most distal 3 cm of the anorectal volume HT for 6 vs 18 months	72	Prevalence of peak toxicity grade at 36 months	SOMAT LENT and CTCAE V2.0	1. No predictive factors identified
Schaake et al. (30)	Prospective cohort	262	IMRT Dose: 78 Gy CTV= prostate only +/− SV; PTV= 10 mm isotropic margin Pelvic floor muscles were defined retrospectively Anal canal = most distal 3 cm of the anorectal volume HT was allowed	> 36 months	Prevalence grade ≥2 toxicities after 36 months	CTCAE V3.0 and patient questionnaire	1. No clinical or dosimetric correlation with rectal pain
Thor et al. (29)	Prospective cohorts	212 in the Danish cohort 277 in the Swedish cohort	3DCRT Dose: 70 to 78 Gy CTV= prostate only +/− SV PTV= For the Danish cohort, 7 mm margin but 9 mm cranio-caudally. For the Swedish cohort, 20 mm margin but 15 mm posteriorly Definition of the anal sphincter (AS) and of external/internal sphincter muscles definition du sphincter anal (AS)	42 months for the Danish cohort 76 months for the Swedish cohort	Prevalence of moderately severe symptoms (occurring at least once/week) and the use of diapers/pads	Questionnaires specific to the Danish and Swedish cohorts	1. HT, tobacco and V15 Gy are predictive of pain

RCT, randomized controlled trial; 3D, tridimensional radiotherapy; RT, radiotherapy; CTV, clinical target volume; PTV, planning target volume; SV, seminal vesicles; HT, hormonotherapy; GIT, gastrointestinal toxicities; OR, odds ratio; HR, hazard ratio.

### Recommendations on How to Optimize the Anal Canal Radio-Induced Tolerance

**Table 5** summarizes all the recommendations derived from this literature review regarding irradiation of the anal canal during prostate RT.

**Table 5:** Optimization of late gastrointestinal tolerance after radiotherapy for localized prostate cancer.

	Delineation of structures
**Recommendations**	Contour the anal canal separately from the anorectal volume The anal canal starts at the anorectal junction, either at the level of the levators or where the rectum starts to angle downwards and posteriorly. It ends at the anal verge (use a radiopaque marker if possible). The pectinous line is at mid-canal, it measures ~3 to 4 cm in height. Pelvic floor muscles, sphincters and pudendal nerves could be identified during contouring
	**Personalization of treatment**
**History of abdominal surgery** **Acute GI symptoms before, during or after RT** **Use of anticoagulants or hormonotherapy** **Elderly patient**	Inform patients on the increased risk of late GIT
**Tobacco and other cardiovascular risk factors**	Tobacco cessation and control of risk factors
**Biomarkers**	Inform patients on the existence of these predictive tests that have not been validated yet in current routine practice

#### Taking Into Account the Patients’ Medical History and Their Individual Factors in Predicting Late GIT

It is primordial to consider the patients’ clinical factors, in addition to the dosimetric analysis, in order to limit RT related LGIT (28). An adaptation of the dose levels could then be done based on these clinical risk factors. Patients should be informed of a potential increased risk of GIT if they have any of the described risk factors, namely any prior abdominal surgery, cardiovascular or smoking history or history of GI symptoms prior to RT. We also believe that the concept of rectal capacitance, which is well described in the context of RT for rectal cancer (39), should be further studied in prostate RT in order to better predict the risk of LGIT. This specific point could be extrapolated to the AC in future studies. An exciting area of innovation is the emergence of biomarkers (genomic, SNPs, micro-RNA, radiation-induced lymphocyte apoptosis, etc.) that can predict radiation induced toxicities. These could allow clinicians to adapt the management of patients that have individual susceptibility to ionizing radiation (40). Indeed, there are reports already of the existence of genetic factors that could predispose to anorectal bleeding (41–44).

#### Contouring the Anal Canal Separately From the Rectum During Treatment Planning

Our literature review showed that there is a significant heterogeneity in the dosimetric studies looking at LGIT. Moreover, the pathophysiology of the AC is unique and independent of that of the rest of the GI system (ano-rectal and/or small bowel). Thus, it is important to contour the AC separately from the rectum or anorectal volume during treatment planning. We propose that an anal probe be inserted in the AC specifically and metal fiducial markers placed at the anal verge at the time of treatment simulation in order to facilitate delineation of the AC. Contouring of the AC should follow published guidelines (4).

#### Considering the Dose-Effect Relationship

Intensity-modulated, image-guided radiotherapy (IMRT/IGRT) was shown to significantly decrease the incidence of GIT and is now the standard of care for prostate RT (45). Thus, it is important to mention that most of the dosimetric data derived from our literature review are from 3DCRT studies. Furthermore, there are no reports in the literature of AC-specific dosimetric data in case of moderate hypofractionated RT for the prostate (46). Taking these caveats into account, our literature review shows that low, medium, and high doses of radiation were all correlated to AC-specific LGIT. This makes it extremely complex to establish dosimetric constraints for the AC that would help in limiting GIT. These findings, however, highlight a possible dose-effect relationship for the AC. To definitively prove such a dose-effect relationship, it will be important in future trials to specifically delineate the AC as an OAR and include an evaluation of the GIT specifically related to the AC.

#### Identifying and Contouring Anatomical Structures Adjacent to the Anal Canal During Treatment Planning

Different muscles and nerves could be individually contoured with the help of MRI imaging, and assuming that they receive similar doses to the AC, the same dose constraints could be applied in order to reduce the risk of LGIT.

#### Evaluation of Toxicities Using Standardized Scales and Quality of Life Questionnaires

One of the main limitations to the evaluation of LGIT, as highlighted in this review, is the heterogeneity that exists among the different symptom evaluation scales used in studies. The use of specific, well-validated symptoms scales is key in the design of future prospective studies evaluating the tolerance of the AC to RT. By the same token, it will be important to include quality of life scales and Patients Reported Outcomes questionnaires in these studies.

#### Use of Spacers and Endorectal Balloons

Studies on the daily use of endorectal balloons showed that they efficiently reduce doses to the AC and the risks of LGIT. Thus, the use of spacer technologies could be a viable technical approach in reducing AC-related GIT (47).

## Limitations of the Study

One of the main limitations of this narrative review is the fact that it is based on a small number of publications (nine). This is mainly because our research question is very specific and that there is limited literature on the specific subject of AC-related LGIT and limited publications that delineated the AC individually in their study design. Another limitation of the study is the fact that the majority of the reviewed papers used 3DCRT, which is no longer the standard of care for the treatment of prostate cancer. Moreover, we did not identify any reports of moderate hypofractionated prostate RT looking specifically at the contribution of AC dosimetry to the development of LGIT. There is also the variability related to the different toxicity scales used throughout studies as well as the definition and contouring of the anal canal subvolumes. Other limitations are inherent to any narrative review, including bias related to the selection of papers, which we tried to mitigate by having specific inclusion and exclusion criteria. However, we recognize that certain publications meeting our inclusion criteria may have been missed. Finally, we want to highlight the fact that a causal relation between dose to the AC and the occurrence of LGIT cannot be inferred from this literature review. The evidence summarized here suggests an important role for the AC, however, this role cannot be fully dissociated from the contribution of the rectum, anorectum, or other adjacent structures and of patient-related predictive factors.

## Conclusion

Despite limited literature on the subject, our review highlighted the potential role that irradiation of the AC plays in the development of LGIT after prostate/pelvic RT. It also highlights how important it is to take patients’ specific clinical risk factors into account. Identification of the AC independently of the anorectal volume, and an optimization of the dosimetry related to the AC and adjacent structures, will be essential in improving side effects related to prostate RT. Finally, tolerance to prostate RT should be evaluated using validated scales and quality of life questionnaires in the context of prospective studies.

## Data Availability Statement

The original contributions presented in the study are included in the article/supplementary material. Further inquiries can be directed to the corresponding author.

## Author Contributions

Conceptualization: PS, MD, RC, and MB. Data curation: PS, MB, and RC. Formal analysis: PS, MD, and RC. Investigation: PS, MD, RC, and MB. Methodology: PS and RC. Project administration: PS and MB. Supervision: PS and RC. Validation: SS, IL, DA, TN, TV, and VV. Visualization: all co-authors. Writing—original draft: PS, MD, and MB. Writing—review and editing: all co-authors. All authors contributed to the article and approved the submitted version.

## Conflict of Interest

The authors declare that the research was conducted in the absence of any commercial or financial relationships that could be construed as a potential conflict of interest.

## References

[1] Gami B Harrington K Blake P Dearnaley D Tait D Davies J. How Patients Manage Gastrointestinal Symptoms After Pelvic Radiotherapy. Aliment Pharmacol Ther (2003) 18(10):987–94. 10.1046/j.1365-2036.2003.01760.x 14616164

[2] Krol R Smeenk RJ van Lin EN Hopman WP. Impact of Late Anorectal Dysfunction on Quality of Life After Pelvic Radiotherapy. Int J Colorectal Dis (2013) 28(4):519–26. 10.1007/s00384-012-1593-5 23080344

[3] Moreau-Claeys MV Peiffert D. Normal Tissue Tolerance to External Beam Radiation Therapy: Anal Canal. Cancer Radiother (2010) 14(4-5):359–62. 10.1016/j.canrad.2010.01.009 20418146

[4] Noel G Antoni D Barillot I Chauvet B. [Delineation of Organs at Risk and Dose Constraints]. Cancer Radiother (2016) 20(Suppl):S36–60. 10.1016/j.canrad.2016.07.032 27516050

[5] Yeoh EE Holloway RH Fraser RJ Botten RJ Di Matteo AC Moore JW. Anorectal Dysfunction Increases With Time Following Radiation Therapy for Carcinoma of the Prostate. Am J Gastroenterol (2004) 99(2):361–9. 10.1111/j.1572-0241.2004.04037.x 15046230

[6] Matta R Chapple CR Fisch M Heidenreich A Herschorn S Kodama RT. Pelvic Complications After Prostate Cancer Radiation Therapy and Their Management: An International Collaborative Narrative Review. Eur Urol (2019) 75(3):464–76. 10.1016/j.eururo.2018.12.003 30573316

[7] Resnick MJ Koyama T Fan KH Albertsen PC Goodman M Hamilton AS. Long-Term Functional Outcomes After Treatment for Localized Prostate Cancer. N Engl J Med (2013) 368(5):436–45. 10.1056/NEJMoa1209978 PMC3742365 23363497

[8] Lund JA Kaasa S Wibe A Widmark A Fransson P. Late Radiation Effects to the Rectum and Anus After Treatment for Prostate Cancer; Validity of the LENT/SOMA Score. Acta Oncol (2013) 52(4):727–35. 10.3109/0284186X.2013.747695 23398595

[9] Yeoh EK Holloway RH Fraser RJ Botten RJ Di Matteo AC Butters J. Pathophysiology and Natural History of Anorectal Sequelae Following Radiation Therapy for Carcinoma of the Prostate. Int J Radiat Oncol Biol Phys (2012) 84(5):e593–9. 10.1016/j.ijrobp.2012.06.032 22836050

[10] Petersen SE Bregendahl S Langschwager M Laurberg S Brock C Drewes AM. Pathophysiology of Late Anorectal Dysfunction Following External Beam Radiotherapy for Prostate Cancer. Acta Oncol (2014) 53(10):1398–404. 10.3109/0284186X.2014.926029 24960583

[11] Yeoh EK Bartholomeusz DL Holloway RH Fraser RJ Botten R Di Matteo A. Disturbed Colonic Motility Contributes to Anorectal Symptoms and Dysfunction After Radiotherapy for Carcinoma of the Prostate. Int J Radiat Oncol Biol Phys (2010) 78(3):773–80. 10.1016/j.ijrobp.2009.08.050 20153122

[12] Choi Y Park W Rhee PL. Can Anorectal Manometry Findings Predict Subsequent Late Gastrointestinal Radiation Toxicity in Prostate Cancer Patients? Cancer Res Treat (2016) 48(1):297–303. 10.4143/crt.2014.333 25779364 PMC4720090

[13] Gulliford SL Foo K Morgan RC Aird EG Bidmead AM Critchley H Evans PM. Dose-Volume Constraints to Reduce Rectal Side Effects From Prostate Radiotherapy: Evidence From MRC RT01 Trial ISRCTN 47772397. Int J Radiat Oncol Biol Phys (2010) 76(3):747–54. 10.1016/j.ijrobp.2009.02.025 19540054

[14] National Cancer Institute (US). Cancer Therapy Evaluation Program. In: Common Terminology Criteria for Adverse Events: (CTCAE). Cancer Therapy Evaluation Program (2003). Available at: https://ctep.cancer.gov/protocoldevelopment/electronic_applications/ctc.htm.

[15] Pan YB Maeda Y Wilson A Glynne-Jones R Vaizey CJ. Late Gastrointestinal Toxicity After Radiotherapy for Anal Cancer: A Systematic Literature Review. Acta Oncol (2018) 57(11):1427–37. 10.1080/0284186X.2018.1503713 30264638

[16] Peeters ST Lebesque JV Heemsbergen WD van Putten WL Slot A Dielwart MF. Localized Volume Effects for Late Rectal and Anal Toxicity After Radiotherapy for Prostate Cancer. Int J Radiat Oncol Biol Phys (2006) 64(4):1151–61. 10.1016/j.ijrobp.2005.10.002 16414208

[17] Peeters ST Hoogeman MS Heemsbergen WD Hart AA Koper PC Lebesque JV. Rectal Bleeding, Fecal Incontinence, and High Stool Frequency After Conformal Radiotherapy for Prostate Cancer: Normal Tissue Complication Probability Modeling. Int J Radiat Oncol Biol Phys (2006) 66(1):11–9. 10.1016/j.ijrobp.2006.03.034 16757129

[18] Cox JD Stetz J Pajak TF. Toxicity Criteria of the Radiation Therapy Oncology Group (RTOG) and the European Organization for Research and Treatment of Cancer (EORTC). Int J Radiat Oncol Biol Phys (1995) 31(5):1341–6. 10.1016/0360-3016(95)00060-C 7713792

[19] Fellin G Fiorino C Rancati T Vavassori V Baccolini M Bianchi C. Clinical and Dosimetric Predictors of Late Rectal Toxicity After Conformal Radiation for Localized Prostate Cancer: Results of a Large Multicenter Observational Study. Radiother Oncol (2009) 93(2):197–202. 10.1016/j.radonc.2009.09.004 19828205

[20] Valdagni R Vavassori V Rancati T Fellin G Baccolini M Bianchi C. Increasing the Risk of Late Rectal Bleeding After High-Dose Radiotherapy for Prostate Cancer: The Case of Previous Abdominal Surgery. Results From a Prospective Trial. Radiother Oncol (2012) 103(2):252–5. 10.1016/j.radonc.2012.03.012 22521747

[21] Fellin G Rancati T Fiorino C Vavassori V Antognoni P Baccolini M. Long Term Rectal Function After High-Dose Prostatecancer Radiotherapy: Results From a Prospective Cohort Study. Radiother Oncol (2014) 110(2):272–7. 10.1016/j.radonc.2013.09.028 24332020

[22] Fiorino C Rancati T Fellin G Vavassori V Cagna E Casanova Borca V. Late Fecal Incontinence After High-Dose Radiotherapy for Prostate Cancer: Better Prediction Using Longitudinal Definitions. Int J Radiat Oncol Biol Phys (2012) 83(1):38–45. 10.1016/j.ijrobp.2011.06.1953 21985939

[23] Buettner F Gulliford SL Webb S Sydes MR Dearnaley DP Partridge M. The Dose-Response of the Anal Sphincter Region–an Analysis of Data From the MRC RT01 Trial. Radiother Oncol (2012) 103(3):347–52. 10.1016/j.radonc.2012.03.002

[24] Fiorino C Fellin G Rancati T Vavassori V Bianchi C Borca VC. Clinical and Dosimetric Predictors of Late Rectal Syndrome After 3D-CRT for Localized Prostate Cancer: Preliminary Results of a Multicenter Prospective Study. Int J Radiat Oncol Biol Phys (2008) 70(4):1130–7. 10.1016/j.ijrobp.2007.07.2354 17881142

[25] Smeenk RJ Hopman WP Hoffmann AL van Lin EN Kaanders JH. Differences in Radiation Dosimetry and Anorectal Function Testing Imply That Anorectal Symptoms may Arise From Different Anatomic Substrates. Int J Radiat Oncol Biol Phys (2012) 82(1):145–52. 10.1016/j.ijrobp.2010.08.023 20950951

[26] Smeenk RJ Hoffmann AL Hopman WP van Lin EN Kaanders JH. Dose-Effect Relationships for Individual Pelvic Floor Muscles and Anorectal Complaints After Prostate Radiotherapy. Int J Radiat Oncol Biol Phys (2012) 83(2):636–44. 10.1016/j.ijrobp.2011.08.007 22137024

[27] Ebert MA Foo K Haworth A Gulliford SL Kennedy A Joseph DJ. Gastrointestinal Dose-Histogram Effects in the Context of Dose-Volume-Constrained Prostate Radiation Therapy: Analysis of Data From the RADAR Prostate Radiation Therapy Trial. Int J Radiat Oncol Biol Phys (2015) 91(3):595–603. 10.1016/j.ijrobp.2014.11.015 25596108

[28] Defraene G Van den Bergh L Al-Mamgani A Haustermans K Heemsbergen W Van den Heuvel F. The Benefits of Including Clinical Factors in Rectal Normal Tissue Complication Probability Modeling After Radiotherapy for Prostate Cancer. Int J Radiat Oncol Biol Phys (2012) 82(3):1233–42. 10.1016/j.ijrobp.2011.03.056 21664059

[29] Thor M Olsson CE Oh JH Petersen SE Alsadius D Bentzen L. Relationships Between Dose to the Gastro-Intestinal Tract and Patient-Reported Symptom Domains After Radiotherapy for Localized Prostate Cancer. Acta Oncol (2015) 54(9):1326–34. 10.3109/0284186X.2015.1063779 PMC4786008 26340136

[30] Schaake W van der Schaaf A van Dijk LV Bongaerts AH van den Bergh AC Langendijk JA. Normal Tissue Complication Probability (NTCP) Models for Late Rectal Bleeding, Stool Frequency and Fecal Incontinence After Radiotherapy in Prostate Cancer Patients. Radiother Oncol (2016) 119(3):381–7. 10.1016/j.radonc.2016.04.005 27157889

[31] Fiorino C Valdagni R Rancati T Sanguineti G. Dose-Volume Effects for Normal Tissues in External Radiotherapy: Pelvis. Radiother Oncol (2009) 93(2):153–67. 10.1016/j.radonc.2009.08.004 19765845

[32] Landoni V Fiorino C Cozzarini C Sanguineti G Valdagni R Rancati T. Predicting Toxicity in Radiotherapy for Prostate Cancer. Phys Med (2016) 32(3):521–32. 10.1016/j.ejmp.2016.03.003 27068274

[33] Drean G Acosta O Ospina JD Fargeas A Lafond C Corrégé G. Identification of a Rectal Subregion Highly Predictive of Rectal Bleeding in Prostate Cancer IMRT. Radiother Oncol (2016) 119(3):388–97. 10.1016/j.radonc.2016.04.023 27173457

[34] Buettner F Gulliford SL Webb S Sydes MR Dearnaley DP Partridge M. Assessing Correlations Between the Spatial Distribution of the Dose to the Rectal Wall and Late Rectal Toxicity After Prostate Radiotherapy: An Analysis of Data From the MRC RT01 Trial (ISRCTN 47772397). Phys Med Biol (2009) 54(21):6535–48. 10.1088/0031-9155/54/21/006 19826203

[35] Loganathan A Schloithe AC Hutton J Yeoh EK Fraser R Dinning PG. Pudendal Nerve Injury in Men With Fecal Incontinence After Radiotherapy for Prostate Cancer. Acta Oncol (2015) 54(6):882–8. 10.3109/0284186X.2015.1010693 25734401

[36] Yeoh E Tam W Schoeman M Moore J Thomas M Botten R. Argon Plasma Coagulation Therapy Versus Topical Formalin for Intractable Rectal Bleeding and Anorectal Dysfunction After Radiation Therapy for Prostate Carcinoma. Int J Radiat Oncol Biol Phys (2013) 87(5):954–9. 10.1016/j.ijrobp.2013.08.034 24113059

[37] van Lin EN Kristinsson J Philippens ME de Jong DJ van der Vight LP Kaanders JH. Reduced Late Rectal Mucosal Changes After Prostate Three-Dimensional Conformal Radiotherapy With Endorectal Balloon as Observed in Repeated Endoscopy. Int J Radiat Oncol Biol Phys (2007) 67(3):799–811. 10.1016/j.ijrobp.2006.09.034 17161552

[38] Cicchetti A Rancati T Ebert M Fiorino C Palorini F Kennedy A. Modelling Late Stool Frequency and Rectal Pain After Radical Radiotherapy in Prostate Cancer Patients: Results From a Large Pooled Population. Phys Med (2016) 32(12):1690–7. 10.1016/j.ejmp.2016.09.018 27720692

[39] Ihnat P Slívová I Tulinsky L Ihnát Rudinská L Máca J Penka I. Anorectal Dysfunction After Laparoscopic Low Anterior Rectal Resection for Rectal Cancer With and Without Radiotherapy (Manometry Study). J Surg Oncol (2018) 117(4):710–6. 10.1002/jso.24885 29094352

[40] Brengues M Lapierre A Bourgier C Pèlegrin A Özsahin M Azria D. T Lymphocytes to Predict Radiation-Induced Late Effects in Normal Tissues. Expert Rev Mol Diagn (2017) 17(2):119–27. 10.1080/14737159.2017.1271715 27936985

[41] Someya M Yamamoto H Nojima M Hori M Tateoka K Nakata K. Relation Between Ku80 and MicroRNA-99a Expression and Late Rectal Bleeding After Radiotherapy for Prostate Cancer. Radiother Oncol (2015) 115(2):235–9. 10.1016/j.radonc.2015.04.008 25937401

[42] Valdagni R Rancati T Ghilotti M Cozzarini C Vavassori V Fellin G. To Bleed or Not to Bleed. A Prediction Based on Individual Gene Profiling Combined With Dose-Volume Histogram Shapes in Prostate Cancer Patients Undergoing Three-Dimensional Conformal Radiation Therapy. Int J Radiat Oncol Biol Phys (2009) 74(5):1431–40. 10.1016/j.ijrobp.2008.10.021 19211196

[43] Kerns SL Fachal L Dorling L Barnett GC Baran A Peterson DR. Radiogenomics Consortium Genome-Wide Association Study Meta-Analysis of Late Toxicity After Prostate Cancer Radiotherapy. J Natl Cancer Inst (2020) 112:179–90. 10.1093/jnci/djz075 PMC7019089 31095341

[44] Burri RJ Stock RG Cesaretti JA Atencio DP Peters S Peters CA. Association of Single Nucleotide Polymorphisms in SOD2, XRCC1 and XRCC3 With Susceptibility for the Development of Adverse Effects Resulting From Radiotherapy for Prostate Cancer. Radiat Res (2008) 170(1):49–59. 10.1667/RR1219.1 18582155

[45] Delobel JB Gnep K Ospina JD Beckendorf V Chira C Zhu J. Nomogram to Predict Rectal Toxicity Following Prostate Cancer Radiotherapy. PLoS One (2017) 12(6):e0179845. 10.1371/journal.pone.0179845 28640871 PMC5480987

[46] Langrand-Escure J de Crevoisier R Llagostera C Créhange G Delaroche G Lafond C. Dose Constraints for Moderate Hypofractionated Radiotherapy for Prostate Cancer: The French Genito-Urinary Group (GETUG) Recommendations. Cancer Radiother (2018) 22(2):193–8. 10.1016/j.canrad.2017.11.004 29628205

[47] Leiker AJ Desai NB Folkert MR. Rectal Radiation Dose-Reduction Techniques in Prostate Cancer: A Focus on the Rectal Spacer. Future Oncol (2018) 14(26):2773–88. 10.2217/fon-2018-0286 29939069

